# Use of Herbal Drugs in Cardiovascular Disease- A Review

**DOI:** 10.2174/011573403X323724240830075719

**Published:** 2024-09-03

**Authors:** Arshdeep Kaur, Ranjeet Kumar

**Affiliations:** 1 Department of Pharmacy Practice, ISF College of Pharmacy, Moga, (Punjab) India

**Keywords:** Cardiovascular disorder (CVD), tumor necrosis factor-a (TNF-a), ACE inhibitory peptides (ACEIPs), interferon regulatory factor 3 (IRF3), coronary heart disease (CHD), nuclear factor-kappaB (NF-kB), arterial blood pressure (ABP)

## Abstract

Thirty percent of deaths worldwide are caused by cardiovascular disorders (CVDs). As per the WHO data, the number of fatalities due to CVDs is 17.9 million years, and it is projected to cause 22.2 million deaths by 2030. In terms of gender, women die from CVD at a rate of 51% compared to 42% for males. Most people use phytochemicals, a type of traditional medicine derived from plants, either in addition to or instead of commercially available medications to treat and prevent CVD. Phytochemicals are useful in lowering cardiovascular risks, especially for lowering blood cholesterol, lowering obesity-related factors, controlling blood sugar and the consequences of type 2 diabetes, controlling oxidative stress factors and inflammation, and preventing platelet aggregation. Medicinal plants that are widely known for treating CVD include ginseng, *Ginkgo biloba*, ganoderma lucidum, gynostemma pentaphyllum, viridis amaranthus, *etc*. Plant sterol, flavonoids, polyphenols, sulphur compound and terpenoid are the active phytochemicals present in these plants. The aim of this article is to cover more and more drugs that are used for cardiovascular diseases. In this article, we will learn about the use of different herbal drugs, mechanism of action, phytochemical compounds, side effects, *etc*. However, more research is required to comprehend the process and particular phytochemicals found in plants that treat CVD.

## 1 INTRODUCTION

Diseases affecting the heart or blood vessels are referred to as cardiovascular diseases (CVDs). A global yearly death toll from CVDs exceeds 17 million. Thus, CVDs continue to be the world's most common causes of mortality, constituting a significant global economic and health burden. According to the World Health Organisation (WHO), 31% of fatalities worldwide occur each year as a result of CVDs [1]. According to the European Cardiovascular Disease Statistics 2017, 45% of fatalities in Europe are attributable to CVDs [2]. Current figures from the American Heart Association indicate that around half of Americans suffer from a cardiovascular disease [3]. Cardiovascular diseases (CVDs) encompass a range of illnesses, such as peripheral arterial disorders, coronary heart disease (CHD), heart failure,myocardial infarction (heart attack), strokes, cardiomyopathies, dyslipidemias, and hypertension [4]. The need for new alternative treatments arises from the chronically increasing incidence of CVD, which highlights the shortcomings of existing therapeutic techniques. Herbal remedies have shown more promise in recent years as complementary treatments for conditions linked to the cardiovascular system [5]. Atherosclerosis and hypertension are the two main causes of cardiovascular disease (CVDs), and herbal extracts and derivatives of them can positively regulate and eventually improve these molecular processes. Due to the abundance of bioactives found in herbal treatments, their cellular methods of action are multimodal [6]. Herbal medicines actually possess anti-inflammatory, anti-proliferative, vasorelaxant, antioxidant, and diuretic properties [7]. Herbal treatments can also stop the phenotypic flipping of VSMCs and decrease ROS generation, platelet activation, endothelial dysfunction, lipid peroxidation, and macrophage atherogenicity. Herbal remedies can be utilised for the treatment and management of a variety of CVDs due to their broad spectrum of biochemical and cellular targets [8]. To treat a variety of CVDs, such as CHD, myocardial infarction, and others, Salvia miltiorrhiza, often known as red sage, is an annual sage that has long been utilised in Chinese medicine. Angina pectoris and atherosclerosis. Mostly, the dried-out root of the Danshen plant rhizome is used to extract the active ingredients. Herbs are frequently utilised in traditional as well as contemporary medicine, but there aren't many reviews that compile them and thoroughly discuss their modes of action and safety is present when it comes to CVDs. Plant-based substances seem to offer protection against cardiovascular disease; nevertheless, the most beneficial compounds are lavonoids, terpenoids, saponins and polysaccharides [9]. Four of the most well-known herbal preparations-*Ginkgo biloba*, *Ganoderma lucidum*, *Gynostemma pentaphyllum*, *Aniba canelilla*, *Aspalathus linearis*, *etc*.-include these extremely potent chemicals, which we will discuss in this review.

## 2 DRUGS OBTAINED FROM HERBAL PLANTS

It would seem that nature is a major source for developing novel medications that aid in the treatment of illnesses. *Salix alba* L. aspirin, *Digitalis purpurea's digoxin* (cardiac glycoside), *Ephedra sinica's ephedrine*, *Monascus purpureus L.'s lovastatin*, *Taxus brevifolia's taxol*, *Rauvolfia serpentina's reserpine*, and several more well-known medications derived from plants and herbs [10]. It's interesting to see that reserpine still works well to treat hypertension [11]. Notably, the identification of antimalarial drugs-such as quinine from the bark of Cinchona species and artemisinin from *Artemisia annua L.-offers* a prime example of how ethnomedicine can guide the creation of novel pharmaceuticals [12]. Among the compounds is papaverine from *Papaver somniferum*, which serves as the foundation for the hypertension drug verapamil and galegine from *Galega officinalis* L., that is utilised in the synthesis of metformin and other bisguanidine-type diabetes medications [13]. A profitable industry exists around the production of pharmaceuticals from natural products like herbs; during the years 1983–1994, natural compounds or their derivatives accounted for 39% of the 520 novel medications approved; during that same period, natural products also accounted for 60–80% of antimicrobial and anticancer drugs [14].

## 3 HERBAL MEDICINE FOR HYPERTENSION AND ATHEROSCLEROSIS MANAGEMENT

The two main factors that increase the risk of CVDs, hypertension and atherosclerosis, can be favourably modulated and eventually improved by herbal remedies and their derivatives. Herbal treatments have multiple cellular modes of action since they contain a variety of bioactives. Herbal treatments can actually have diuretic, anti-inflammatory, vasorelaxant, antioxidant, and anti-proliferative properties. Inhibiting endothelial dysfunction, activation of platelets, lipid peroxidation, and production of ROS & microglia atherogenicity are additional benefits of using herbal treatments to stop VSMC phenotypic flipping. Herbal remedies can be utilised for the treatment and management of a variety of CVDs due to their broad spectrum of molecules as well as cellular targets. For example, *Salvia miltiorrhiza*, also referred to as red sage, is an annual sage that is commonly used in Chinese medicine to treat a range of CVDs, including coronary heart disease myocardial infarction (MI), atherosclerosis, and angina pectoris [15].

## 4 GINSENG

The herb ginseng was first grown 2000 years ago, in part because of its ceremonial use [16]. Asia, including China, Japan, Korea, and Vietnam, as well as North America, including the USA and Canada, are home to ginseng. The ginsengs that are used most commonly are Japanese ginseng (the *Panax japonicas* C.A. Mey.), the ginseng from America (*Panax quinquefolium* L.), Chinese ginseng (Panax species *Notoginseng Burkill*; by F.H. Chen.), and Korean red ginseng (KRG; the species *Panax ginseng* C.A. Mey.). The roots of plants that are 5 to 7 years old are usually either dried in the air in the sun to produce white ginseng and steam-treated for two to three hours at 98 to 100°C before being sunshine-dried to produce red ginseng. Red ginseng has more pharmacological potency than white ginseng due to chemical changes that occur during steaming [17]. These days, ginseng is either prepared and consumed in liquid form (such as tea or oil extracts) or in solid form (such as pills, capsules, or dried roots) [14]. On the other hand, it has been consistently shown that ginseng root, berry, and leaf extracts have anti-obesity, anti-hyperglycemic, anti-hypertensive, insulin sensitization, and anti-hyperlipidemic properties [16]. From ginseng, over three hundred bioactives have been identified. Triterpene saponins called ginsenosides are the main bioactive components found in ginseng preparations [18]. The most researched ginsenosides of the 40 that have been identified so far are Rb1, Rg1, Rg3, Re, and Rd. The red Korean ginseng is unusual in terms of Rg3, Rg5, and RK1 [19]. The study of ginseng and its components has become so popular that a publication called The Publication of Ginseng Research (https://www.journals.elsevier.com/journal-of-ginseng-research) is presently devoted to the subject. Lately, there has been an increase in interest in the purified individual ginsenosides as opposed to the whole ginseng root extract. Vasorelaxation, anti-oxidation, anti-inflammatory, and anti-cancer properties are exhibited by ginseng and its ginsenoside components [20].

## 5 GINSENG AT THE BENCH: MECHANISM OF ACTION IN CVDS

Ginseng has been utilized to treat hypertension in relation to CVDs. Ginseng's ability to enhance vascular function results in its hypotensive effects. It's interesting to note that ginsenosides promote vasorelaxation in many vessels: the aortas of rats [21], coronary arteries in mice [20], and the cerebral arteries of monkeys [22]. While ginsenoside Rg3 stimulates eNOS, ginseng can boost NO generation and eNOS expression [14]. Vascular tone is improved by KRG's induction of NO-dependent vasorelaxation. The augmentation of eNOS dimer formation, the rise in NO production, and the suppression of arginase activity work together to mediate these effects [23]. Research has demonstrated that the G115 extract of Panax ginseng inhibits both angiotensin I-induced stiffness of bovine mesenteric arteries and ACE activity in the endothelial cells of human umbilical veins (HUVECs) [24]. Additional benefits of ginseng for managing CVDs include its anti-oxidant [25], anti-inflammatory [26], and anti-hyperlipidemic [27] effects, in addition to controlling Ca^2+^ channels [27].

Inhibiting ACE activity, blocking Ca^2+^-gated channels, activating Ca^2+^-gated potassium channels, and raising NO and cGMP levels are all possible with ginsenoside Rg3 [28]. Additionally, ginseng has been shown to have anti-inflammatory properties by blocking the activation of nuclear factor-kappaB (NF-kB) and activator protein (AP-1), which in turn lowers the production of COX-2, IL-6, IL-1b, and tumor necrosis factor-a (TNF-a) [26]. Baek *et al.* in macrophages showed that every component of the KRG has a distinct mechanism by which it exerts anti-inflammatory effects. The expression of inflammatory genes such as iNOS, COX-2, TNF a, and interferon-b was lower and NO generation was dramatically decreased by the saponin fraction, for example. By contrast, all extracts, including those containing water, saponin, and non-saponin fractions, reduce the transcriptional activity of its downstream effector interferon regulatory factor 3 (IRF3) as well as the activity of the kinase TAK1 [29]. Dietary supplementation with KRG decreases blood cholesterol levels and lessens the development of atherosclerotic lesions caused by a high cholesterol diet in rabbits by preventing diacylglycerol liberation [30]. Once more, the saponin fraction of *P. notoginseng* may attenuate cholesterol esters in amaebocytes via upregulating the adenosine triphosphate-binding cassette transporterA1 [31]. Furthermore, ginseng has demonstrated a strong *in vivo* antithrombotic impact; this effect may be attributable to an antiplatelet action rather than an anticoagulant activity, suggesting that ginseng use may be advantageous for people with elevated risks of thrombosis and cardiovascular diseases [19]. Within this framework, it has been revealed that the dihydro-ginsenoside Rg3 can potently reduce platelet aggregation by modulating downstream intracellular signals, including cAMP and extracellular signal-regulated kinase 2 [32].

## 6 SAFETY, TOXICITY, AND SIDE EFFECTS OF GINSENG

After prolonged high dosages of ginseng extracts were administered, very few adverse effects were seen. These included diarrhea in the morning, skin eruptions, anxiety, insomnia, edema, hypertension, reduced appetite, depression, and hypotension. According to Lee *et al*., *P. ginseng* extract given at a dose of 1 or 2 g/day for 4 weeks was safe, palatable, and had no harmful effects on healthy volunteer subjects. Haematological and biochemical tests only showed non-significant alterations [33]. Numerous cases of extended or improper ginseng usage have been reported, which may have contributed to potential cardiovascular side effects like elevated blood pressure [34], atrial fibrillation, also known as long QT syndrome. For instance, a young man's three-year ginseng intake has been linked to hypertension, dyspnea, and symptoms such as lightheadedness and difficulty focusing that vanished and did not return after the supplements were stopped.

Although ginseng has been shown to interact with a number of medications, the most well-researched interaction is with the blood thinner warfarin [35]. Ginsenosides were found to dramatically increase the activity of two enzymes known to metabolise warfarin, P450 CYP3A4 and P450 CYP2C9, in a recent study conducted on rats. This resulted in the restoration of the levels of coagulation factors II and VII as well as the protein Z, which are typically inhibited by warfarin [36].

## 7 GINKGO BILOBA

*Ginkgo biloba*, one of the oldest seed plants, is also called the maidenhair tree in English because of its likeness to the leaf of the maidenhair fern. Considered a “living fossil” because it has survived for 270 million years without experiencing any significant alterations [37]. Its origin is thought to be in the Yangtze River Valley in eastern China [38].

## 8 MODE OF ACTION

*Ginkgo biloba* enhances the lifespan of cardiomyocytes and prevents their apoptosis via regulating the PI3K-AKT and nuclear factor-kB pathways [39].

## 9 SAFETY, TOXICOLOGY, AND ADVERSE REACTIONS OF *G. BILOBA*

When used orally at the recommended dosage, GBE may have moderate side effects, the most typical of which are headache, dizziness, constipation, mild gastrointestinal distress, and allergic skin responses. However, higher amounts can cause weakness, nausea, vomiting, diarrhoea, and restlessness [40]. There is a temporal correlation between GBE consumption and major bleeding episodes, such as cerebral bleeding, according to fifteen published case studies [41], a result that could be explained by the extract's other ingredients, such as ginkgolides and bilobalides, which inhibit platelet-activating factor [42]. Most of these severe bleeding incidents, such as subarachnoid and cerebral haemorrhages, have been connected to the concurrent use of ginkgo and antiplatelet and/or anticoagulant drugs [43]. It is, therefore, advised to cease using GBE a minimum of two weeks before surgery. Due to their anti-platelet characteristics, GBEs (including seeds and leaves) have always been advised to be taken with caution during lactation and throughout pregnancy, especially in the vicinity of labour [44].

## 10 GANODERMA LUCIDUM

Ganoderma lucidum, sometimes referred to as “lingzhi” or “reishi,” is a mushroom whose mycelia, spores, and fruit body are utilised to manufacture various commercial versions of *G. Lucidum* for their therapeutic advantages. Commercially, *G. Lucidum* is offered in a variety of forms, including supplements, tea, and powders. The bioactive components found in *G. Lucidum* are diverse and comprise triterpenes, carbohydrates, nucleosides, minerals, proteins, peptides, amino acids, fatty acids, alkaloids, and steroids [45].

## 11 MODE OF ACTION

Antioxidants play a critical role in preventing atherosclerosis, as previously mentioned. As an antioxidant, *G. lucidum* has been shown to protect Caenorhabditis elegans, the model organism, due to oxidative stress caused by heavy metals and paraquats by blocking the mTOR/S6K signalling pathway and imposing dietary restrictions, respectively [46]. It has been discovered that *G. lucidum* guards against oxidative damage caused by hydrogen peroxide to human lymphocyte DNA [47]. *G. lucidum* ethanol extract improved the phosphorylation of Nrf_2_, which elevated HO^-1^ in C_2_C_12_ myoblasts. It was also shown that *G. lucidum* controls the manifestation of Nrf_2_, which in turn controls antioxidant genes like HO^-1^, GST, and NQO^-1^ [48]. Consequently, *G. lucidum's* antioxidant capacity, which is crucial for preventing atherosclerosis, can influence lots of signalling channels.

Increased blood pressure may be harmful to cardiac health. *G. lucidum* yielded three peptides known as ACE inhibitory peptides (ACEIPs): QLVP, QDVL, and QLDL. These peptides have the ability to inhibit ACE activity and are utilised in the treatment of hypertension. Through its interactions with ACE's Gln242 and Lys472, QLVP can block ACE. In HUVECs, QLVP also decreased the mRNA and protein production of the vasoconstrictor peptide endothelin-1 and increased the phosphorylation of eNOS mediated by Angiotensin 1 [49].

To gain a more thorough understanding of how G. Lucidum bioactive derivatives function, additional research must be done on the potential processes and signalling pathways that these compounds use to elicit their therapeutic effects.

## 12 SAFETY, TOXICITY, AND SIDE EFFECTS OF *G. LUCIDUM*

After three to six months, this herb may cause skin allergies that lead to dry skin and have an impact on some organs, including: mouth, throat, and nose. Additional allergy symptoms include headaches, dizziness, rashes, upset stomachs, excessive bleeding, and bloody stools. When using Ganoderma lucidum, those with extremely elevated or decreased blood pressure are more likely to experience adverse effects. Some studies state that taking powdered Ganoderma powder has caused liver damage or chronic diarrhoea in some patients. Taking excessive doses of Ganoderma can raise the possibility of bleeding if you have a low platelet count. It is not advised for females who are nursing or pregnant to take this mushroom. However, giving healthy human volunteers a dose of 1.5 g/d of *G. lucidum* extract for four weeks had no effect on hemostatic parameters, platelet counts, or general hemostatic functions. Furthermore, there were not any bleeding issues in the healthy patients after using *G. lucidum* [50].

## 13 GYNOSTEMMA PENTAPHYLLUM

*Gynostemma pentaphyllum*, a herbaceous climbing vine, is often referred to as Jiaogulan. Widely available throughout South and East Asia, having originated in South China [51].

## 14 MODE OF ACTION

Shows synergistic effects by raising oxidation and decreasing lipid synthesis. Additionally, it exhibits protection against the development of foam cells and intracellular lipid accumulation brought on by oxidised low-density lipoprotein. Finally, it regulates cholesterol metabolism [52].

## 15 SAFETY, TOXICITY & SIDE EFFECT OF G. PENTAPHYLLUM

*G. pentaphyllum* is a non-toxic material that has no negative effects, even when consumed in large quantities. Rats were given up to 750 mg/kg of *G. pentaphyllum* over an extended period of time without experiencing any toxicity or mortality. Rats exposed to standardised *G. pentaphyllum* water extract did not exhibit any toxicity [53]. For two months, each of the three groups of healthy subjects received 50, 200, or 400 mg twice daily of a water extract of *G. pentaphyllum* as part of a Phase I clinical trial aimed at assessing the safe use of the botanical specimen. Significant changes in the amount of the CD^3+^, CD^4+^, and CD^8+^ cells, or in the activity of natural killer cells, were not described as serious immunological adverse events. There was also no discernible change in any biochemical measures. *G. pentaphyllum* dosages such as these were considered safe [54].

### 15.1 Gracian foxglove

**Phytochemical compounds:** Steroid glycoside and digoxin

**Mode of action:** protects cardiac hypertrophy, oxidative stress, apoptosis, increased release of atrial natriuretic peptide, and KATP channel opening from cardiomyocytes. Safe guarding for macrophages and monocytes. (i) Therapeutically protective effects are achieved by the nuclear receptors a peroxisome proliferator-activated receptors α activation, suppression of the NOS-NO signalling pathway, and activation of the oestrogen receptor.

(i) Through preventing, modifying, and controlling the creation of structural, contractile, and glycoprotein proteins; (ii) By controlling calcium concentrations and enhancing mitochondrial function [77].

### 15.2 Geneciana

**Phytochemical compounds:** Gentiopicroside, swertiamarin, sweroside, gentiana scabra, gentiana triflora, isogentisin, bellidifolin, a compound called mangiferin, isoorientin, and isovitexin.

**Mode of action:** For the treatment of thromboembolic illnesses, amarogentin may hold therapeutic promise because it inhibits platelet activation (i) by phosphorylating the MAPK pathway and the phospholipase C(PLC)2-PKC cascade. Amarogentin may have therapeutic promise for the treatment of thromboembolic disorders since (ii) via blocking the influx and release of Ca^2+^ from intracellular reserves stops and cures atherosclerosis.

Through preventing venous smooth muscle cell development.(i) KCl, caffeine, and norepinephrine significantly reduce the contracturant response; (ii) norepinephrine preconstricts the aortic rings, acting as an endothelium-independent vasodilator [78].

### 15.3 Marsdenia latifolia

**Family:** Apocynacea

Parts of plant: Leaves

**Phytoconstituents:** Phenolic compounds, saponins, triterpenes, branched glycosylated, oleanane saponins, furostane saponin, rutin, kaempferol, and iridoid ebuloside.

**Mode of action:** lowering blood pressure (i) by managing the heart rate and (ii) preventing cardiac contraction (iii) regulate high heart rate and mean arterial pressure (iv); by enhancing cardiac output and ultimately lowering arterial blood pressure (ABP) (v); and by blocking the channel for calcium ions [79].

### 15.4 Gynostemma spp.

**Family:** Cucurbitaceae

**Section of plants:** leaves

**Phytoconstituents:** Gypenosides and saponins of the dammarane type

**Mode of action:** H9c2 embryonic cardiomyocytes are protected against I/R damage (i) By reducing intracellular ROS generation and restoring normal levels of mitochondrial membrane potential (ii) Through the PI3K/Akt pathway being activated. Reduction of cell death in response to I/R stimuli by (i) inhibiting the homologous proteins pathway and (ii) down regulating the expression of proteins known to cause cell death, such as caspase-3 and bax and (iii) preventing endoplasmic reticulum stress-induced apoptosis [52].

## 16 OTHER MEDICINAL PLANTS USE IN CARDIOVASCULAR DISEASE

Phalaenopsis moldavica, Eleocharisdulcis, Pentaphyllum gynostemma, olifera moringa, stenopetala moringa, Nigella sativa, the highest sanctum, Olive europaea, Panax species, American Persea, Dactylifera phoenix, Potentilla spp., Rose Rhodiola, Damascene Rosa, obtusifolius Rumex, Trifolium vulgare, Miltiorrhiza salvia, The Lycopersicum Solanum, Mollusca sophora, Myrtillus vaccine, Magnesium grandiflorum, Folidium viburnum, Zingibe oleraceum, Curcuma prolonga, Gentiana species, latifolium gongronema, Astypus spp., oleifera moringa, Sativa Nigella, Glabratus Oncocalyx, and Marianum Silymarin are used [80-83].

## CONCLUSION

Globally, the main cause of death is CVDs. Herbal drugs are widely used for CVDs as they have fewer adverse effects and are less expensive than synthetic medications. For a very long time, phytochemicals have been used to treat this disease. Particularly when it comes to lowering blood cholesterol levels, obesity risk factors, glucose regulation and diabetes type 2 effects, oxidative stress factor regulation, platelet aggregation inhibition, and inflammatory regulation, phytochemicals are useful in lowering cardiovascular risks. The processes by which phytochemicals exert their cardio-protective benefits are multifaceted and mostly still unknown. More extensive research is still needed to fully understand the pharmacological characteristics of medicinal plants' phytochemicals, including their effects on the cardiovascular system, despite the preclinical and clinical studies that have been conducted suggesting a positive correlation among their intake and a decreased level of cardiovascular factors. According to available data, these herbal remedies have strong therapeutic qualities and can improve pathological states connected to cardiovascular diseases. Nevertheless, definitive clinical therapeutic advantages are still pending. Therefore, more thorough clinical research is needed to determine the safety, toxicity, and effectiveness of phytochemicals in therapeutic plants. Therefore, it is not safe to suggest these herbal remedies as a substitute for conventional medicinal medication. Actually, several of these herbs' toxicity and safety have recently given rise to possible worries (*i.e*. *Gingko Biloba*).

## Figures and Tables

**Table 1:** Significant phytochemicals have been documented to help treat heart conditions.

**Plant's Scientific Name**	**Sections of the Plant**	**Phytochemical Substances**	**Actions or Uses**	**References**
*Viridis amaranthus* Family: Amarnth	Entire plant section	The amino acid	Manages high cholesterol.	[55]
*Aniba elliptica* *Family: laurus L.*	Leaf, bark, and stem wood Stem	Phenolic acids, flavonoids, and essential oil	Controls the oxidative damage and bp.	[56]
*Rooibos* *Family: Legume*	Leaflet	Polyphenols	Controls inflammatory response and oxidative responses.	[57]
*Carqueja* *Family: asteraceae*	Aerial segment	Compounds phenolic and flavonoids	Due to its ability to reduce lipid levels and prevent the development of free radicals.	[58]
*Berberis vulgaris L* *Family: Berberidaceae*	Root, Bark, and Stem	Berberina	Controls oxidative responses, immunity, and metabolism.	[59]
*Camellia biflora* *Family: Theaceae*	Leaflet	Saponins and terpenoids	Causes the inhibition of reactive oxygen species after activating the the route of bradykinin-NO, which in turn causes cardioprotection against ischemia-reperfusion injury.	[60]
*Camellia oleosa* *Family: Theaceae*	Leave	Alkaloids, terpenoids, sterols, and catechins	Reduces the amounts of total cholesterol as well as low-density lipoprotein cholesterol, and nonhigh-density lipoprotein cholesterol, apolipoprotein B, and these substances.	[61]
*Calcitrapa tinctoria Roehl.* *Family: Asteraceae*	Floweret	Chalcone molecules	Lower renin activity and bp.	[62]
*Citrus bergamia* *Family: Rue or citrus family*	Fruits	Flavonoid	Controls cardiotoxicity because to its lipid-lowering and antioxidant properties.	[63]
*Calappa nucifera* *Family: Arecaceae*	Flower, leaves, seeds, and stem	Alkaloids, triterpenes, flavonoids, phenols, tannins, and leucoanthocyanidins	Reduces oxidative response and damage to cells.	[64]
*Cilantro* *Family: Apiaceae*	Seed	Essential oil and polyphenol	Assists diabetics with dyslipidemia in avoiding heart problems.	[65]
*Saffron crocus* *Family: Iridaceae*	Stigma of flowers	Crocin, crocetin, picrocrocin, and essential oil	Demonstrates positive effects against atherosclerosis and hypertension.	[66]
*Maclura tricuspidata carrier* *Family: Moraceae*	Leave	Vital oil	Lowers hypertension patients' and *systolic blood pressure (SBP)*.	[67]
*Curcuma domestica* *Family: Zingiberaceae*	Rootstalk	Diferuloylmethane	Benefits for the heart by lowering oxidative stress.	[68]
*Moldavian dragonhead* Family: deadnettle or sage family	Seedbed	Flavonoid substances: Rosmarinic acid, luteolin, and tianin	Lowers the amounts of TNF-α and IL-1β while strengthening the myocardial fibres and membrane.	[69]
*Andropogon dulcis* *Family: Cyperaceae*	Fruits	Sugars, phenolics, sterols, saponins, and amino acids	Maintains cardiovascular health in a state of equilibrium.	*[* *70* *]*
*Thouars* *Family: Clusiaceae*	Fruits	Flavonoids and phenolic compounds together	Shielding the heart from harm to the myocardium	[71]
*Ginkyo* *Family: Ginkgoaceae*	Leafs	Terpenoids and flavonoids	Enhances cardiomyocyte viability and impedes cardiomyocyte death via regulating the PI3K-AKT and NF-κB pathways.	[39]
*Soya bean* *Family: Angiospermae*	Seed	Isoflavones: The glycoside form of glycitin, genistein, and daidzein	Enhances resistance to oxidation of low-density lipoprotein, boosts vascular reactivity, and prevents thrombus formation.	[72]
*Jiaogulan* *Family: Cucurbitaceae*	Aerial parts	Saponin	Exhibits synergistic actions in reducing lipid production as well as in increasing oxidation; it also shows prevention against development of foam cells and intracellular lipid buildup caused by oxidised low-density lipoprotein. It also regulates cholesterol metabolism.	[52]
*Murunga* *Family: Moringaceae*	Leafs	Saponin	Lowers bp and blood cholesterol levels.	[73]
*African Moringa* *Family: Moringaceae*	Leafs	Heart glycosides, terpenoids, polyphenols, alkaloids, and saponins	Treats hypertension.	[74]
*Nigella Family: Ranunculaceae*	Seed	Cinnamaldehydes	Reduces oxidative responses and controls to prevent the development of hypertension.	[75]
*Ocimum tenuiflorum* *Family: Lamiaceae*	Leafs	Eugenols	Demonstrates the substantial impact of reducing blood pressure on treating cardiovascular consequences.	[76]
*African wild olive* *Family: Oleaceae*	Leafs	Iridoid and secoiridoid	Plays a cardioprotective role by preventing the oxidation of fat and the gathering of cholesterol.	[74]

## LIST OF ABBREVIATIONS

CHD: Coronary Heart Disease
CVDs: Cardiovascular Diseases
WHO: World Health Organisation

## References

[1] Ndejjo R. (2021). Cardiovascular disease prevention in Mukono and Buikwe districts in Uganda: evidence to implementation.. Doctoral thesis, University of Antwerp,.

[2] Martinet W., Coornaert I., Puylaert P., De Meyer G.R.Y. (2019). Macrophage death as a pharmacological target in atherosclerosis.. Front. Pharmacol..

[3] Benjamin E.J., Muntner P., Alonso A. (2019). Heart disease and stroke statistics—2019 update: a report from the American Heart Association.. Circulation.

[4] Reiner Ž., Laufs U., Cosentino F., Landmesser U. (2019). The year in cardiology 2018: prevention.. Eur. Heart J..

[5] Chen L., Fu G., Hua Q. (2022). Efficacy of add-on Danhong injection in patients with unstable angina pectoris: A double-blind, randomized, placebo-controlled, multicenter clinical trial.. J. Ethnopharmacol..

[6] Tagde P., Tagde P., Tagde S. (2021). Natural bioactive molecules: An alternative approach to the treatment and control of glioblastoma multiforme.. Biomed. Pharmacother..

[7] Aggarwal B.B. (2011). Identification of novel anti-inflammatory agents from Ayurvedic medicine for prevention of chronic diseases:‘reverse pharmacology’ and ‘bedside to bench’ approach.. Curr. Drug Targets.

[8] Ruparelia N., Chai J.T., Fisher E.A., Choudhury R.P. (2017). Inflammatory processes in cardiovascular disease: a route to targeted therapies.. Nat. Rev. Cardiol..

[9] Shaito A., Thuan D.T.B., Phu H.T. (2020). Herbal medicine for cardiovascular diseases: efficacy, mechanisms, and safety.. Front. Pharmacol..

[10] Harvey A. (2000). Strategies for discovering drugs from previously unexplored natural products.. Drug Discov. Today.

[11] Weber M.A., Schiffrin E.L., White W.B. (2014). Clinical practice guidelines for the management of hypertension in the community a statement by the American Society of Hypertension and the International Society of Hypertension.. J. Hypertens..

[12] Cragg G.M., Newman D.J. (2013). Natural products: A continuing source of novel drug leads.. Biochim. Biophys. Acta, Gen. Subj..

[13] Fabricant D.S., Farnsworth N.R. (2001). The value of plants used in traditional medicine for drug discovery.. Environ. Health Perspect..

[14] Valli G., Giardina E.G.V. (2002). Benefits, adverse effects and drug interactionsof herbal therapies with cardiovascular effects.. J. Am. Coll. Cardiol..

[15] Gao S., Liu Z., Li H., Little P.J., Liu P., Xu S. (2012). Cardiovascular actions and therapeutic potential of tanshinone IIA.. Atherosclerosis.

[16] Kim J.H. (2012). Cardiovascular diseases and Panax ginseng: a review on molecular mechanisms and medical applications.. J. Ginseng Res..

[17] Kim W.Y., Kim J.M., Han S.B. (2000). Steaming of ginseng at high temperature enhances biological activity.. J. Nat. Prod..

[18] Mahady G.B., Gyllenhaal C., Fong H.H.S., Farnsworth N.R. (2000). Ginsengs: a review of safety and efficacy.. Nutr. Clin. Care.

[19] Lee C.H., Kim J.H. (2014). A review on the medicinal potentials of ginseng and ginsenosides on cardiovascular diseases.. J. Ginseng Res..

[20] Pan SY (2013). New perspectives on how to discover drugs from herbal medicines: CAM’s outstanding contribution to modern therapeutics.
Evid based Compl Altern Med.

[21] Kim N.D., Kang S.Y., Kim M.J., Park J.H., Schini-Kerth V.B. (1999). The ginsenoside Rg3 evokes endothelium-independent relaxation in rat aortic rings: role of K+ channels.. Eur. J. Pharmacol..

[22] Toda N., Ayajiki K., Fujioka H., Okamura T. (2001). Ginsenoside potentiates NO-mediated neurogenic vasodilatation of monkey cerebral arteries.. J. Ethnopharmacol..

[23] Shin W., Yoon J., Oh G.T., Ryoo S. (2013). Korean red ginseng inhibits arginase and contributes to endothelium-dependent vasorelaxation through endothelial nitric oxide synthase coupling.. J. Ginseng Res..

[24] Persson I.A.L., Dong L., Persson K. (2006). Effect of Panax ginseng extract (G115) on angiotensin-converting enzyme (ACE) activity and nitric oxide (NO) production.. J. Ethnopharmacol..

[25] Lee H., Hong Y., Tran Q. (2019). A new role for the ginsenoside RG3 in antiaging via mitochondria function in ultraviolet-irradiated human dermal fibroblasts.. J. Ginseng Res..

[26] Keum Y.S., Han S.S., Chun K.S. (2003). Inhibitory effects of the ginsenoside Rg3 on phorbol ester-induced cyclooxygenase-2 expression, NF-κB activation and tumor promotion.. Mutat. Res..

[27] Park M.Y., Lee K.S., Sung M.K. (2005). Effects of dietary mulberry, Korean red ginseng, and banaba on glucose homeostasis in relation to PPAR-α, PPAR-γ, and LPL mRNA expressions.. Life Sci..

[28] Kim N.D., Kang S.Y., Park J.H., Schini-Kerth V.B. (1999). Ginsenoside Rg3 mediates endothelium-dependent relaxation in response to ginsenosides in rat aorta: role of K+ channels.. Eur. J. Pharmacol..

[29] Baek K.S., Yi Y.S., Son Y.J. (2016). In vitro and *in vivo* anti-inflammatory activities of Korean Red Ginseng-derived components.. J. Ginseng Res..

[30] Hwang S.Y., Son D.J., Kim I.W. (2008). Korean red ginseng attenuates hypercholesterolemia‐enhanced platelet aggregation through suppression of diacylglycerol liberation in high‐cholesterol‐diet‐fed rabbits.. Phytother. Res..

[31] Jia Y., Li Z.Y., Zhang H.G., Li H.B., Liu Y., Li X.H. (2010). Panax notoginseng saponins decrease cholesterol ester via up-regulating ATP-binding cassette transporter A1 in foam cells.. J. Ethnopharmacol..

[32] Lee W.M., Kim S.D., Park M.H. (2010). Inhibitory mechanisms of dihydroginsenoside Rg3 in platelet aggregation: Critical roles of ERK2 and cAMP.. J. Pharm. Pharmacol..

[33] Lee N.H., Yoo S.R., Kim H.G., Cho J.H., Son C.G. (2012). Safety and tolerability of Panax ginseng root extract: a randomized, placebo-controlled, clinical trial in healthy Korean volunteers.. J. Altern. Complement. Med..

[34] Coon J.T., Ernst E. (2002). Panax ginseng.. Drug Saf..

[35] Yuan C.S., Wei G., Dey L. (2004). Brief communication: American ginseng reduces warfarin’s effect in healthy patients: a randomized, controlled Trial.. Ann. Intern. Med..

[36] Dong H., Ma J., Li T. (2017). Global deregulation of ginseng products may be a safety hazard to warfarin takers: solid evidence of ginseng-warfarin interaction.. Sci. Rep..

[37] Hori T., Ridge R.W., Tulecke W., Del Tredici P., Trémouillaux-Guiller J., Tobe H. (2012). Ginkgo biloba a global treasure: from biology to medicine..

[38] Jaggy H., Koch E. (1997). Chemistry and biology of alkylphenols from Ginkgo biloba L.. Pharmazie.

[39] Xiang K., Yang J., Wu X., Peng J., Guo J., Fan C. (2021). Advances in traditional Chinese medicine for cardiovascular disease therapy in 2020.. Traditional Medicine Research.

[40] Diamond B.J., Bailey M.R. (2013). Ginkgo biloba.. Psychiatr. Clin. North Am..

[41] Bent S., Goldberg H., Padula A., Avins A.L. (2005). Spontaneous bleeding associated with Ginkgo biloba.. J. Gen. Intern. Med..

[42] Izzo A.A., Ernst E. (2009). Interactions between herbal medicines and prescribed drugs: an updated systematic review.. Drugs.

[43] Matthews M.K. (1998). Association of Ginkgo biloba with intracerebral hemorrhage.. Neurology.

[44] Dugoua J-J., Mills E., Perri D., Koren G. (2006). Safety and efficacy of ginkgo (Ginkgo biloba) during pregnancy and lactation.. Can. J. Clin. Pharmacol..

[45] Ahmad M.F. (2018). Ganoderma lucidum: Persuasive biologically active constituents and their health endorsement.. Biomed. Pharmacother..

[46] Cuong V.T., Chen W., Shi J. (2019). The anti-oxidation and anti-aging effects of Ganoderma lucidum in Caenorhabditis elegans.. Exp. Gerontol..

[47] Shi Y., James A.E., Benzie I.F.F., Buswell J.A. (2002). Mushroom‐derived preparations in the prevention of H 2 O 2 ‐induced oxidative damage to cellular DNA.. Teratog. Carcinog. Mutagen..

[48] Yoon H-M., Lee Y., Kim J. (2016). Ethanol extract of Ganoderma lucidum augments cellular anti-oxidant defense through activation of Nrf2/HO-1.. J. Pharmacopuncture.

[49] Wu Q., Li Y., Peng K. (2019). Isolation and characterization of three antihypertension peptides from the mycelia of Ganoderma lucidum (Agaricomycetes).. J. Agric. Food Chem..

[50] Kwok Y., Ng K.F.J., Li C.C.F., Lam C.C.K., Man R.Y.K. (2005). A prospective, randomized, double-blind, placebo-controlled study of the platelet and global hemostatic effects of Ganoderma lucidum (Ling-Zhi) in healthy volunteers.. Anesth. Analg..

[51] Li Y., Lin W., Huang J., Xie Y., Ma W. (2016). Anti-cancer effects of Gynostemma pentaphyllum (thunb.) makino (jiaogulan).. Chin. Med..

[52] Nguyen N.H., Ha T.K.Q., Yang J.L., Pham H.T.T., Oh W.K. (2021). Triterpenoids from the genus Gynostemma: Chemistry and pharmacological activities.. J. Ethnopharmacol..

[53] Chiranthanut N., Teekachunhatean S., Panthong A., Khonsung P., Kanjanapothi D., Lertprasertsuk N. (2013). Toxicity evaluation of standardized extract of Gynostemma pentaphyllum Makino.. J. Ethnopharmacol..

[54] Chavalittumrong P. (2007). A phase I trial of Gynostemma pentaphyllum Makino in healthy volunteers.. Songklanakarin J. Sci. Technol..

[55] Sarveswaran R., Jayasuriya W., Suresh T.S. (2017). In vitro assays to investigate the anti-inflammatory activity of herbal extracts a review.. World J. Pharmaceut. Res..

[56] Souza-Junior F.J.C., Luz-Moraes D., Pereira F.S. (2020). Aniba canelilla (Kunth) mez (Lauraceae): A review of ethnobotany, phytochemical, antioxidant, anti-inflammatory, cardiovascular, and neurological properties.. Front. Pharmacol..

[57] Smith C., Swart A. (2018). Aspalathus linearis (Rooibos) – a functional food targeting cardiovascular disease.. Food Funct..

[58] Souza M.M.Q., Silva G.R., Cola I.M. (2020). Baccharis trimera (Less.) DC: an innovative cardioprotective herbal medicine against multiple risk factors for cardiovascular disease.. J. Med. Food.

[59] Neag M.A., Mocan A., Echeverría J. (2018). Berberine: Botanical occurrence, traditional uses, extraction methods, and relevance in cardiovascular, metabolic, hepatic, and renal disorders.. Front. Pharmacol..

[60] Singh D., Chaudhuri P.K. (2018). Structural characteristics, bioavailability and cardioprotective potential of saponins.. Integr. Med. Res..

[61] Fürst R., Zündorf I. (2014). Plant-derived anti-inflammatory compounds: hopes and disappointments regarding the translation of preclinical knowledge into clinical progress.. Mediators Inflamm..

[62] Zhou DD (2021). Antioxidant food components for the prevention and treatment of cardiovascular diseases: effects, mechanisms, and clinical studies..

[63] Anwar M.A., Al Disi S.S., Eid A.H. (2016). Anti-hypertensive herbs and their mechanisms of action: part II.. Front. Pharmacol..

[64] Maiuolo J., Carresi C., Gliozzi M. (2021). Effects of bergamot polyphenols on mitochondrial dysfunction and sarcoplasmic reticulum stress in diabetic cardiomyopathy.. Nutrients.

[65] Mounika S., Jayaraman R., Jayashree D. (2021). A comprehensive review of medicinal plants for cardioprotective potential.. Int J Adv Pharm Biotechnol.

[66] Prachayasittikul V., Prachayasittikul S., Ruchirawat S., Prachayasittikul V. (2018). Coriander (Coriandrum sativum): A promising functional food toward the well-being.. Food Res. Int..

[67] Xin L.T., Yue S.J., Fan Y.C. (2017). Cudrania tricuspidata: an updated review on ethnomedicine, phytochemistry and pharmacology.. RSC Advances.

[68] Wang Z., Mu W., Li P., Liu G., Yang J. (2021). Anti-inflammatory activity of ortho-trifluoromethoxy-substituted 4-piperidione-containing mono-carbonyl curcumin derivatives in vitro and in vivo.. Eur. J. Pharm. Sci..

[69] Hesari M., Mohammadi P., Khademi F. (2021). Current advances in the use of nanophytomedicine therapies for human cardiovascular diseases.. Int. J. Nanomedicine.

[70] Zhang Y., Xu H., Hu Z. (2022). Eleocharis Dulcis corm: phytochemicals, health benefits, processing and food products.. J. Sci. Food Agric..

[71] Purohit B.M., Kharadi G.B., Patel K.J., Baxi S., Tripathi C.B. (2016). Evaluation of cardioprotective effect of aqueous extract of Allium cepa Linn. bulb on isoprenaline-induced myocardial injury in Wistar albino rats.. Res. Pharm. Sci..

[72] Vasanthi H.R. (2012). ShriShriMal N, Das DK. Retraction Notice: Phytochemicals from plants to combat cardiovascular disease.. Curr. Med. Chem..

[73] Adekanmi A.A., Adekanmi S.A., Adekanmi O.S. (2020). Qualitative and quantitative phytochemical constituents of moringa leaf.. Int J Eng Inf Syst.

[74] Castejón M.L., Montoya T., Alarcón-de-la-Lastra C., Sánchez-Hidalgo M. (2020). Potential protective role exerted by secoiridoids from Olea europaea L. in cancer, cardiovascular, neurodegenerative, aging-related, and immunoinflammatory diseases.. Antioxidants.

[75] Shamlan G. (2021). Ethanolic and aqueous extracts of avocado (Persea americana) seeds attenuates doxorubicin-induced cardiotoxicity in male albino rats.. Arab. J. Sci. Eng..

[76] Mohammadi Pour P., Farzaei M.H., Soleiman Dehkordi E., Bishayee A., Asgary S. (2021). Therapeutic targets of natural products for the management of cardiovascular symptoms of coronavirus disease 2019.. Phytother. Res..

[77] Shah S.M.A., Akram M., Riaz M., Munir N., Rasool G. (2019). Cardioprotective potential of plant-derived molecules: a scientific and medicinal approach.. Dose Response.

[78] Jiang M., Cui B.W., Wu Y.L., Nan J.X., Lian L.H. (2021). Genus Gentiana: A review on phytochemistry, pharmacology and molecular mechanism.. J. Ethnopharmacol..

[79] Beshel J.A., Palacios J., Beshel F.N. (2020). Blood pressure-reducing activity of Gongronema latifolium Benth. (Apocynaeceae) and the identification of its main phytochemicals by UHPLC Q-Orbitrap mass spectrometry.. J. Basic Clin. Physiol. Pharmacol..

[80] Soonthornkalump S., Chuenboonngarm N., Soontornchainaksaeng P., Jenjittikul T., Thammasiri K. (2014). Effect of colchicine incubation time on tetraploid induction of Kaempferia rotunda.. Acta Hortic..

[81] Hartady T., Syamsunarno M.R.A.A., Priosoeryanto B.P., Jasni S., Balia R.L. (2021). Review of herbal medicine works in the avian species.. Vet. World.

[82] Bachheti R.K., Worku L.A., Gonfa Y.H. (2022). Prevention and treatment of cardiovascular diseases with plant phytochemicals: a review.. Evid. Based Complement. Alternat. Med..

[83] Göttng K.J. (1980). Origin and relationships of the Mollusca.. J. Zool. Syst. Evol. Res..

